# *Bartonella quintana* Deploys Host and Vector Temperature-Specific Transcriptomes

**DOI:** 10.1371/journal.pone.0058773

**Published:** 2013-03-12

**Authors:** Stephanie Abromaitis, Christopher S. Nelson, Domenic Previte, Kyong S. Yoon, J. Marshall Clark, Joseph L. DeRisi, Jane E. Koehler

**Affiliations:** 1 Microbial Pathogenesis and Host Defense Program, University of California San Francisco, San Francisco, California, United States of America; 2 Division of Infectious Diseases, Department of Medicine, University of California San Francisco, San Francisco, California, United States of America; 3 Department of Biochemistry and Biophysics, University of California San Francisco, San Francisco, California, United States of America; 4 Biomedical Sciences Graduate Program, University of California San Francisco, San Francisco, California, United States of America; 5 Department of Veterinary and Animal Science, University of Massachusetts at Amherst, Amherst, Massachusetts, United States of America; 6 Howard Hughes Medical Institute, University of California San Francisco, San Francisco, California, United States of America; University of Louisville, United States of America

## Abstract

The bacterial pathogen *Bartonella quintana* is passed between humans by body lice. *B. quintana* has adapted to both the human host and body louse vector niches, producing persistent infection with high titer bacterial loads in both the host (up to 10^5^ colony-forming units [CFU]/ml) and vector (more than 10^8^ CFU/ml). Using a novel custom microarray platform, we analyzed bacterial transcription at temperatures corresponding to the host (37°C) and vector (28°C), to probe for temperature-specific and growth phase-specific transcriptomes. We observed that transcription of 7% (93 genes) of the *B. quintana* genome is modified in response to change in growth phase, and that 5% (68 genes) of the genome is temperature-responsive. Among these transcriptional changes in response to temperature shift and growth phase was the induction of known *B. quintana* virulence genes and several previously unannotated genes. Hemin binding proteins, secretion systems, response regulators, and genes for invasion and cell attachment were prominent among the differentially-regulated *B. quintana* genes. This study represents the first analysis of global transcriptional responses by *B. quintana*. In addition, the *in vivo* experiments provide novel insight into the *B. quintana* transcriptional program within the body louse environment. These data and approaches will facilitate study of the adaptation mechanisms employed by *Bartonella* during the transition between human host and arthropod vector.

## Introduction

In the last three decades, there has been a resurgence of *Bartonella quintana* infections, with the most severe illness occurring among immunocompromised people [1]. *B. quintana* is a vector-borne Gram-negative bacterium; the vector is the human body louse (*Pediculus humanus humanus*) [2]. In a recent analysis, 33.3% of body lice recovered from infested homeless individuals in California had PCR-detectable *B. quintana* DNA, underscoring the high prevalence of this potentially fatal bacterium in the human environment [3]. *B. quintana* bacteria colonize the louse alimentary tract and establish a life-long commensal relationship within the gut of the body louse, enabling a single louse to infect multiple humans [4]. After introduction into the human host, *B. quintana* can persist in the normally sterile bloodstream for weeks or months [5]. This remarkable, prolonged persistence in the host bloodstream demonstrates the ability of *B. quintana* to avoid clearance by the host immune defenses [6]. Persistent *B. quintana* infections manifest in humans as relapsing fever, endocarditis, and potentially fatal vascular proliferative lesions.

During the infectious cycle, *B. quintana* alternates between two environmental niches: the bloodstream of the human host and the gut of the body louse vector. One important difference between these two niches is the ambient temperature: 37°C in the human bloodstream, and approximately 28°C within the louse gut [7]. To maintain the transmission cycle, *B. quintana* must rapidly deploy the appropriate growth programs to survive and proliferate in the two different environments of host and vector. Modification of the bacterial transcriptome in response to temperature change has been documented in the vector-borne human pathogens *Borrelia burgdorferi* [8], *Yersinia pestis* [9], *Francisella tularensis* [10], and *Rickettsia spp*. [11], [12], [13]. Temperature shift experiments have provided powerful insight into the adaptation of virulence and metabolic programs necessary for niche-specific infection with these vector-borne pathogens [8], [9], [10], [12], [13].

The response and adaptation of *B. quintana* to the distinct niches it occupies has not been studied using global transcriptional analysis. To define the *B. quintana* host- and vector-specific transcriptomes, we designed the first *B. quintana* whole genome DNA microarray (printed by Agilent Technologies, Santa Clara, CA). The array contains 60-mer oligos representing protein coding regions, intergenic regions, and RNA genes. The coverage is approximately six oligos per gene, yielding highly sensitive transcriptional analysis.

In this study, we used the *B. quintana* array to identify growth phase-specific genes and genes that are differentially expressed at host (37°C) and vector (28°C) temperatures. We determined that transcription of 7% (93 genes) of the *B. quintana* genome is modified in response to entry into stationary/death phase, and that 5% (68 genes) of the genome is temperature-responsive. Additionally, analysis of *B. quintana* transcription in infected body lice demonstrated that genes that are highly transcribed at 28°C *in vitro* also were highly transcribed *in vivo*, in the body louse. The temperature-specific genes we identified included type 4 secretion system (T4SS) components, members of the hemin binding protein family, and several previously unannotated genes. Collectively, these temperature-specific genes provide a model for the transcriptional program of the *B. quintana* transition from arthropod vector to human host.

## Materials and Methods

### Bacterial strains and growth conditions

For all experiments, low-passage *B. quintana* wild type strain JK31 was used. The JK31 strain was isolated from a patient co-infected with *B. quintana* and HIV [14]. JK31 *B. quintana* bacteria were streaked onto fresh chocolate agar plates [14] from frozen stock and were grown for 6–7 days in candle extinction jars at 35°C, prior to passage and use in experiments. The liquid media used for *B. quintana* was M199S, which consists of M199 supplemented with 20% fetal bovine serum, glutamine, and sodium pyruvate [15]. For microarray transcription profiling experiments, *B. quintana* JK31 strain bacteria that had been passed once since plating from frozen stock were harvested from 2 confluent chocolate agar plates and resuspended in M199S to a final concentration of 0.6 at OD_600_. 100 µl of the bacterial suspension was plated on each chocolate agar plate. Plates were grown in candle extinction jars at 37°C for 48 h, and then a portion of the jars were shifted to 28°C to grow for the remainder of the temperature shift experiment. A total of 12 biological samples were profiled from two independent time courses.

### *B. quintana* genomic DNA, RNA, and cDNA preparation from bacteria grown *in vitro* on chocolate agar plates, for reverse transcriptase-quantitative PCR (RT-qPCR) and microarray analysis

*B. quintana* genomic DNA was isolated using the Qiagen Puregene Core Kit B (Qiagen, Valencia, CA) following the manufacturer's instructions. For RNA isolation, *B. quintana* were harvested from confluent plates into 1 ml stop solution (M199, 45% EtOH, 5% water-saturated phenol) to prevent RNA degradation [16]. Bacteria were then pelleted by centrifugation at 4,000 × g at 4°C. The bacterial pellet was stored at −80°C until RNA was isolated. Bacterial cells were lysed by incubating in fresh lysozyme (0.4 mg ml^−1^ in 10 mM Tris, 1 mM EDTA) for 5 min at room temperature. The RNA was extracted using TRIzol reagent (Invitrogen, Carlsbad, CA) according to the manufacturer's instructions. Total RNA was RQ1 DNase (Promega, Madison, WI) treated for 3 h and then further purified using the Qiagen RNeasy*®* Mini Kit. For RT-qPCR analysis, cDNA was generated from 0.5 µg of total RNA using random hexamer primers (Invitrogen) and SuperScript™ III (Invitrogen) following the manufacturer's instructions. Reverse transcription reactions without Superscript™ III were performed as negative controls and to evaluate DNase treatment efficiency.

### cDNA generation and labeling for microarray hybridization from *B. quintana* grown *in vitro* on agar, at different temperatures

For microarray analysis, cDNA was generated from 15 µg of total RNA. RNA was combined with 15 µg random nonamer primers (Integrated DNA Technology, San Jose, CA) and 1.8 µl of A/T biased amino-allyl mix in a total of 30 µl. A/T biased amino-allyl mix consisted of 100 mM dATP, 100 mM dGTP, 100 mM dCTP, 100 mM dTTP, and 50 mM amino allyl-dUTP at a ratio of 5∶2.5∶2.5∶1∶8. cDNA reactions were incubated for 5 min at 65°C and then incubated for a minimum of 1 min on ice. 30 µl of reverse transcription mix consisting of Invitrogen reagents (12 µl 5x reverse transcription buffer, 3 µl 0.1 M DTT, 3 µl RNaseOUT, 3 µl SuperScript™ III, and 4.2 µl H_2_O) was added to each reaction. The reactions were incubated for 12 min at 25°C and then for 8 h at 46°C. An additional 3 µl of SuperScript™ III were added to each reaction and the reactions were incubated for an additional 8 h at 46°C. cDNA generation was terminated by a 5 min incubation at 85°C. Reactions were chilled on ice and then treated with RNaseA to degrade remaining RNA. The cDNA was purified using Zymo Research (Irvine, CA) DNA Clean & Concentrator™-25 Kit, following the manufacturer's instructions. Amino-allyl cDNA aliquots were coupled to Cy5 and Cy3 (GE Health Sciences, Piscataway, NJ) in 1.0 M pH 9.0 sodium bicarbonate for 1 h, and then cleaned up with Zymo Research DNA Clean & Concentrator™-25 Kit.

### Genome-wide transcriptional profiling for *B. quintana* grown *in vitro* on agar, at different temperatures

A custom microarray with 15,744 probes was designed using the *B. quintana* Toulouse genomic sequence deposited at NCBI (NC_005955.1), and the annotations found at the Microbial Genome Database for Comparative Analysis (http://mbgd.genome.ad.jp/htbin/MBGD_gene_list.pl?spec=bqu) and JCVI (http://cmr.jcvi.org/cgi-bin/CMR/GenomePage.cgi?org=ntbq01) [17]. Gene sequences were extracted from the genomic sequence with nibFrag Software (http://hgwdev.cse.ucsc.edu/~kent/src/unzipped/utils/nibFrag/, Jim Kent, University of California, Santa Cruz). 60 mer probes were chosen with ArrayOligoSelector software (http://arrayoligosel.sourceforge.net/) with up to 10 oligos per gene. Arrays were ordered in 8×15 K format from Agilent Technologies (Santa Clara, CA) (design amaID 025396).

Hybridization mix was comprised of 10 µl of Cy5 labeled sample, 10 µl of Cy3 labeled pooled reference sample, 5 µl blocking buffer, and 25 µl of Agilent Gene Expression Buffer. Hybridizations were carried out at 65°C for 16–19 h in Agilent hybridization chambers rotating at 10 rpm in a convection oven. After hybridization, the arrays were washed with Agilent wash buffers following the manufacturer's instructions. Image data were acquired taking care to balance the observed total fluorescence in the Cy3 and Cy5 channels. Images were scanned on a Genepix 4000B scanner (Molecular Devices, Union City, CA) and data were extracted using Genepix6.0 software in the Center for Advanced Technology (CAT) at University of California, San Francisco.

### Microarray analysis of *B. quintana* grown *in vitro* on agar, at different temperatures

Raw array data were uploaded to Nomad v2.0 (http://ucsf-nomad.sourceforge.net/), where the data were normalized in bins of pixel intensity R^2^, and then filtered to remove spots with ‘bad’ or ‘missing’ manual flags added during gridding, and spots with sum of median intensities less than 1000. The resulting ratio Cy5/Cy3 intensity tables were log_2_ transformed and re-centered at 0. The log_2_ transformed data were then analyzed using cluster 3.0 (Eisen Laboratory, University of California, Berkeley) and SAM (SAM version 3.0, http://www-stat.stanford.edu/~tibs/SAM/) [18]. SAM results were reported as a ranked list of d-scores that represent the difference between two groups of array data compared to a background of randomly shuffled data with associated false discovery rates (fdr%). The GEO array data accession number is GSE42685, and the array design record is GPL16349.

### Annotation of unannotated temperature-responsive *B. quintana* genes

We translated the open reading frames of gene models with annotations of ‘hypothetical gene’ and ran a blastp query against the nr database with expect threshold 1 and word size of 3. We submitted the same sequences to pHMMER (http://hmmer.janelia.org/search/phmmer), querying against the nr database with sequence E-value cutoff 0.01 and hit E-value cutoff of 0.01, and the default gap-open penalty of 0.02 and gap extension penalty of 0.4.

### Motif search upstream of temperature-regulated *B. quintana* genes

To identify potential *cis* elements involved in the observed temperature response, motif searches with MEME were performed on the list of growth phase- and temperature-regulated genes. The promoters of all the genes in the differentially-regulated lists were taken from the *B. quintana* Toulouse strain genome using Mochiview (http://johnsonlab.ucsf.edu/mochi.html) [19], and then submitted to the motif search algorithm MEME. MEME (http://meme.sdsc.edu/meme/cgi-bin/meme.cgi) searches looked for any number of repetitions of motifs within a sequence for motifs from 6 to 11 bases in length within all of the genes, 37°C up-regulated genes, 28°C up-regulated genes, and the top 11 28°C up-regulated genes.

### Quantitative gene expression analysis by RT-qPCR, from *B. quintana* grown *in vitro* on agar, at different temperatures

For verification of microarray results, RT-qPCR was performed using a MX3000P machine (Stratagene/Agilent Technologies, Santa Clara, CA) to determine the relative abundance of specific mRNA. cDNA was diluted 1∶19 for use in reactions. The reaction mixture included: 10 µL SYBR Fast qPCR master mix (Kapa Biosystems, Woburn, MA), 0.4 µl ROX low (Kapa Biosystems), 7.6 µl template, and 2 µl 1 pmol µl^−1^ primer. The reaction conditions were: 95°C for 10min, 40 cycles of 95°C for 15 s and 60°C for 60 s, with dissociation protocol. Threshold fluorescence was determined during the geometric phase of logarithmic gene amplification; from this, the quantification cycle (C_q_) was set. Standard curves for each primer set were generated by plotting log genomic DNA vs. C_q_. These plots were used to ensure that equivalent reaction efficiency was obtained with all primer sets. Primers used are listed in Table S1. The relative level of gene transcript in samples was determined by converting transcript level into genomic copy number using standard curves. This value was divided by the genomic copy number of the constitutively expressed *B. quintana* reference gene *purA* (adenylosuccinate synthetase) or 16S rRNA, to obtain a relative level of transcription for each gene. Data from three independent experiments were used for statistical analysis by Student's *t* test and to determine average gene transcription values.

### Infection of body lice with *B. quintana*

The body louse (*Pediculus humanus humanus*) strain SF was collected in San Francisco, CA. Collected lice were maintained on human blood using an *in vitro* rearing system [20]. The lice were maintained under conditions of 30°C, 70–80% relative humidity, and light-dark cycles of 16L:8D in a rearing chamber. The human blood (American Red Cross, Dedham, MA) used for feeding was comprised of 25 ml fresh red blood cells (blood type A+) and 25 ml plasma (blood type A+), supplemented with 25 µl of a penicillin plus streptomycin antibiotic mixture (10,000 U penicillin and streptomycin 10 mg per ml, in 0.9% NaCl) [20]. Prior to infection, lice were fed blood without antibiotic supplementation for 2–3 days.

*B. quintana* strain JK31 was used in the body lice infections. Bacteria were harvested from chocolate agar plates, washed with PBS, and then resuspended in human blood without antibiotics for the infection, at a final concentration of 5.77×10^8^±1.20×10^8^ bacteria per ml blood. Female SF strain lice were starved 8 h prior to infection, to ensure feeding on the *B. quintana*-inoculated blood. The lice were fed for 24 h on infected blood or control blood, to which PBS without bacteria had been added. Throughout the remainder of the experiment, lice were fed on uninfected human blood. Populations of uninfected and infected lice were removed from the colony and snap frozen in liquid nitrogen immediately after 24 hours of feeding on the *B. quintana*-containing blood (1 day post-inoculation [dpi]), or 5 days after the commencement of feeding (5 dpi), or 9 days after the commencement of feeding (9 dpi).

### Quantification of *B. quintana* genomic DNA from body lice using real-time PCR, and quantification of *B. quintana* RNA from body lice using reverse transcriptase-quantitative PCR (RT-qPCR)

For genomic *B. quintana* DNA isolation, lice were homogenized in ATL buffer (Qiagen) using a glass Dounce homogenizer. The homogenate was digested with Proteinase K for 16 h at 56°C and then treated with RNaseA. The DNA was then isolated using a Qiagen DNeasy Blood and Tissue kit following the manufacturer's instructions. The number of *B. quintana* bacteria per louse was determined using the isolated genomic DNA as template for real-time PCR. The C_q_ value was used to calculate the DNA copy number by comparison to standard curves. The number of amplified DNA copies was converted into the number of *B. quintana* bacteria assuming 1 attomole gDNA = 3.01×10^5^ cells [21]. Primers used for bacterial quantification are listed in Table S1.

For *B. quintana* RNA isolation from infected lice, lice were homogenized in RLT buffer using a glass Dounce homogenizer and then treated with lysozyme. The sample was then further homogenized using QIAshredder™ columns (Qiagen) following the manufacturer's instructions. RNA was purified from the homogenate using a Qiagen RNeasy*®* Mini Kit following the manufacturer's instructions. The purified RNA was used as template for cDNA synthesis following the protocol above.

## Results and Discussion

### A cluster of growth phase-specific genes is identified in *B. quintana* grown *in vitro* on agar, at either 37°C or 28°C

**Figure 1:**
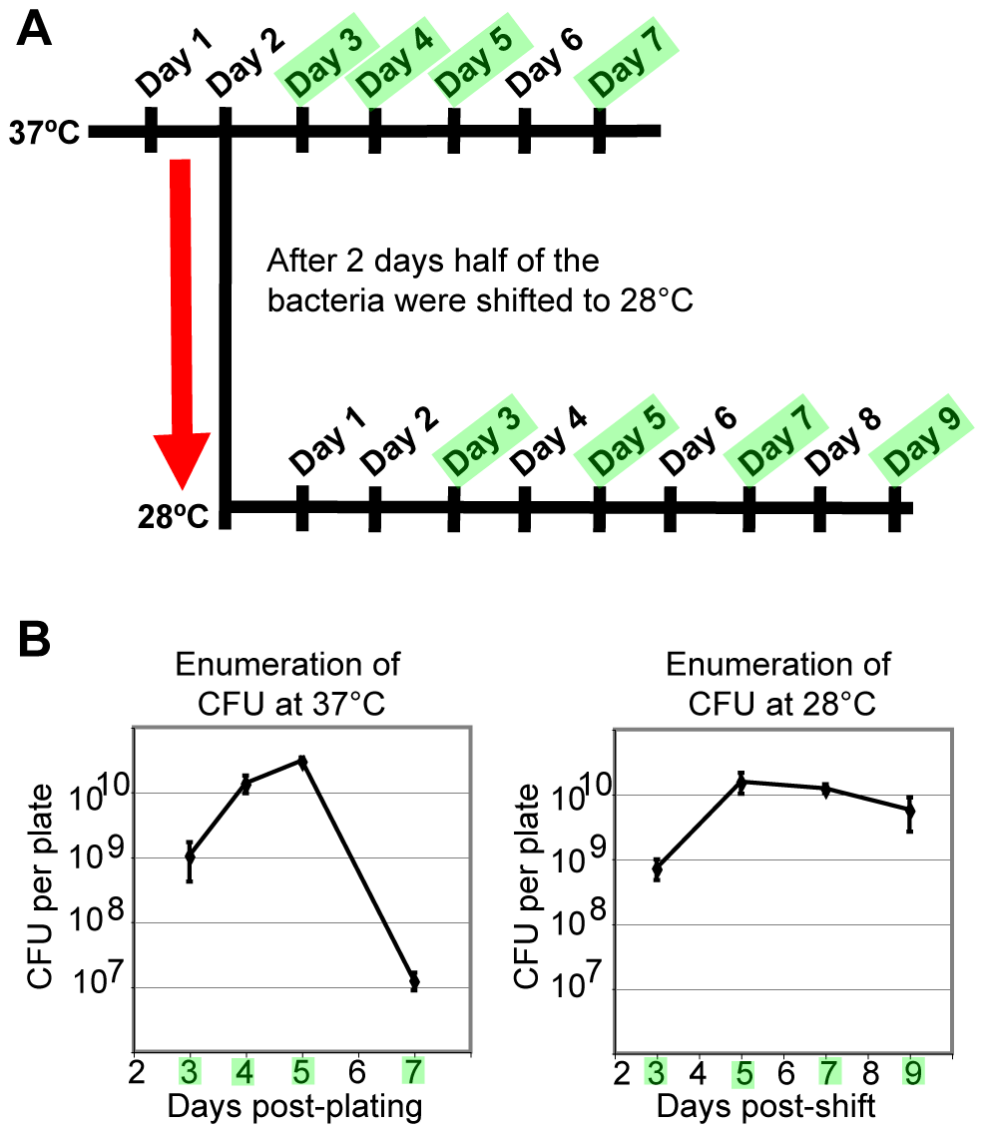
*B. quintana* were enumerated to select time points for microarray analysis of growth stage-regulated genes. **(A)** The diagram depicts the experimental design utilized in cultivation of *B. quintana* for *in vitro* transcriptome profiling at early *vs*. late stage growth. *B. quintana* were plated on chocolate agar and grown at 37°C for 2 days, at which point half of the cultures were shifted to 28°C. *B. quintana* were harvested for RNA extraction and colony-forming unit (CFU) enumeration on the days highlighted in green. **(B)** For each experiment, *B. quintana* growth was analyzed by enumerating CFU per plate after 3 to 11 total days of growth. CFU enumeration was done to determine the growth stage of the *B. quintana* cultures. Based on the data shown in 1B, the days highlighted in green in 1A and 1B were selected for *B. quintana* transcriptional profiling. CFU data from a single representative experiment are shown, and error bars represent the standard deviation of the mean CFU per plate from three replicates.

To ensure that *B. quintana* cultures were in the same phase of growth at 37°C and 28°C, it was first necessary to develop a reproducible and growth stage-matched experimental scheme. Agar-grown cultures of *B. quintana* were synchronized at the two different temperatures, as shown in Figure 1A. Agar media was used for the analysis because we found insufficient growth of *B. quintana* in liquid culture at 28°C. To identify *B. quintana* growth phase-specific genes, bacteria grown at 37°C or 28°C were harvested after 3 to 7 days or 5 to 9 days, respectively (Figure 1A). At each time point, replicate plates were harvested for colony-forming unit (CFU) enumeration.

At 28°C, *B. quintana* demonstrated a brief period of exponential growth on agar, followed by a prolonged stationary phase; death phase was not observed over the 9 days of growth at 28°C (Figure 1B). At 37°C, *B. quintana* exhibited active growth (log phase) 3, 4, and 5 days after plating on solid agar; this was followed by a rapid death phase (Figure 1B). We did not observe a sustained stationary phase at 37°C. Prior to our analysis, *B. quintana* growth dynamics had not been analyzed at the vector temperature of 28°C in any culture medium, but growth of *B. quintana* in liquid media at 37°C or 35°C had been reported by several groups [22], [23], [24]. Similar to our results for agar-grown *B. quintana* at 37°C, cultivation of *B. quintana* in liquid media at 35°C or 37°C resulted in a rapid decrease in CFU per ml following the exponential growth phase, with no detectable stationary phase [22], [23], [24].

**Figure 2:**
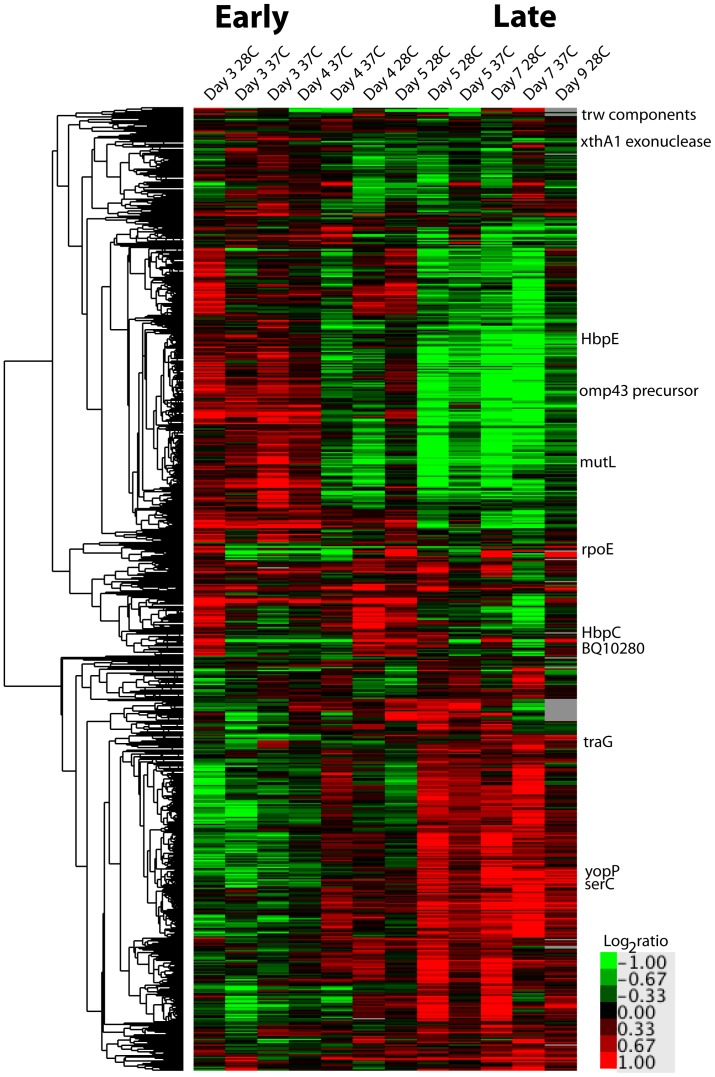
Growth stage-responsive genes comprise two large clusters and include a large proportion of the genome. The heat map depicts unsupervised clustering of data from expression arrays from two independent time courses of *B. quintana* grown at either 28°C or 37°C for 7–9 days, as outlined in Figure 1. The arrays are depicted in columns, and the rows represent the probes on the array. The dendrogram at the left describes the similarity of the gene clusters. Regardless of the temperature at which the *B. quintana* were grown, there is a clear division into two distinct transcriptional programs (genes turned on then off, and off then on, over the duration of the time course). The inset legend shows the range of log_2_-fold changes related to the range of colors in the heatmap. Genes of interest are noted along the right-hand side of the heatmap, in their cluster position.

Analysis of the *B. quintana* transcriptional profile over time at 28°C and 37°C identified both growth stage- and temperature-responsive *B. quintana* genes. We determined that transition from active growth to stationary or death phase elicits a specific transcriptional profile, independent of the temperature at which the *B. quintana* is cultivated (Figure 2). In stationary/death phase, global SAM analysis of transcription identified 10 genes with significantly increased transcription and 83 genes with decreased transcription (changes over 2 fold displayed in Table S2). Growth phase-specific virulence gene regulation has been well documented in a number of bacteria [25], [26], [27], and our *B. quintana* cultures exhibited a robust phase-specific response encompassing 93 significantly altered transcripts. Several of the stationary/death phase responsive genes we identified are associated with *Bartonella* virulence (Figure 2). Among the *B. quintana* virulence genes that were up-regulated during logarithmic phase relative to stationary phase are components of the Trw T4SS (Figure 2). The Trw T4SS in *B. henselae* has been implicated in mediating host-specific erythrocyte adhesion [28], and is likely important for initial colonization of the mammalian host bloodstream by *Bartonella*. A cue provided by the growth phase could prepare the *B. quintana* bacteria for interaction with host erythrocytes after introduction into the host.

### *B. quintana* has unique transcriptional profiles when grown *in vitro* on agar, at human host (37°C) compared with arthropod vector (28°C) temperature

**Figure 3:**
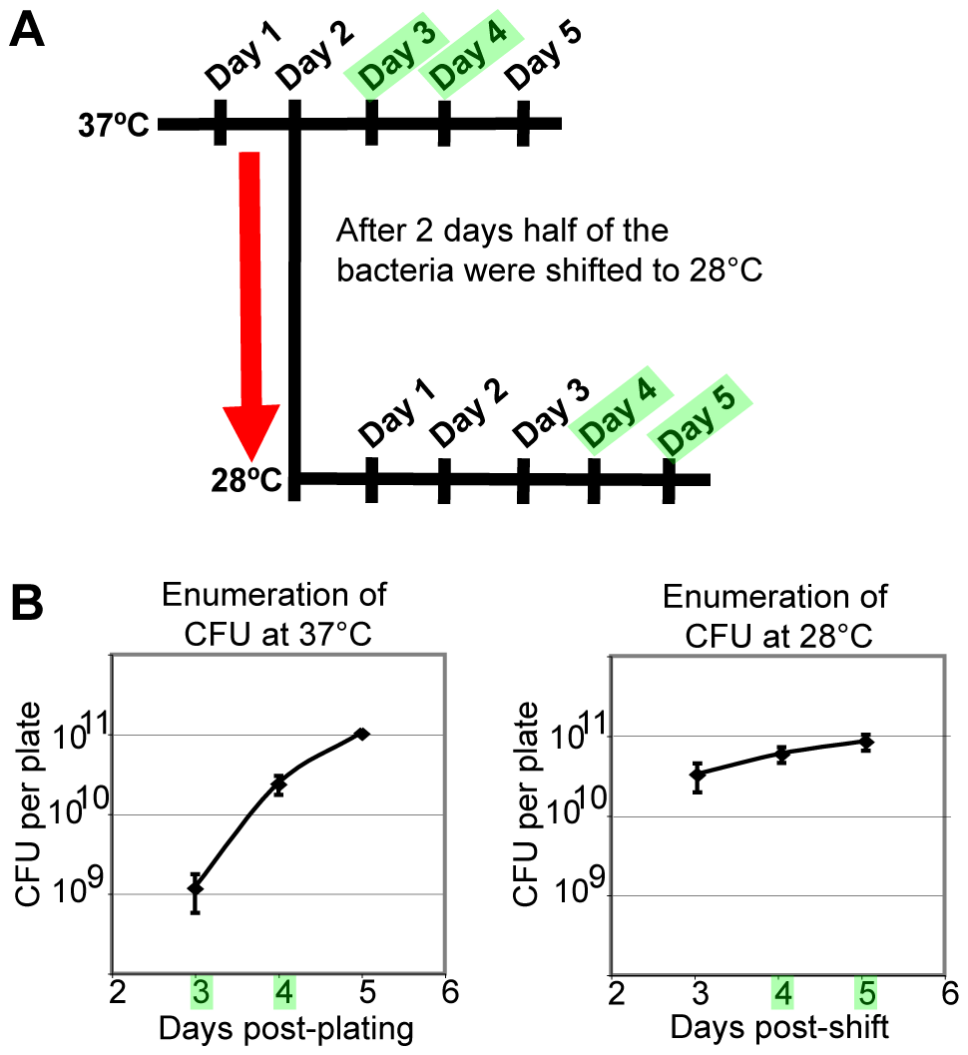
*B. quintana* were enumerated to select time points for microarray analysis of temperature-regulated genes. **(A)** The diagram summarizes the experimental design utilized in cultivation of *B. quintana* for *in vitro* transcriptome profiling at 37°C vs. 28°C. *B. quintana* were plated on chocolate agar and grown at 37°C for 2 days, at which point half of the cultures were shifted to 28°C. *B. quintana* were harvested for RNA extraction and colony-forming unit (CFU) enumeration on the days highlighted in green. **(B)** For each experiment, bacterial growth was analyzed by enumerating CFU per plate from 3 to 7 total days post plating. CFU enumeration was done to ensure that *B. quintana* cultures selected for global transcriptional profiling were in log phase growth at the respective temperatures. The days subsequently selected for *in vitro* transcriptional analysis of *B. quintana* are highlighted in green. CFU data from a single representative experiment are shown, and error bars indicate the standard deviation of the mean CFU per plate from three replicates.

During the infectious cycle, *B. quintana* occupies the bloodstream of the human host and the alimentary tract of the body louse vector. Global transcription in *B. quintana* cultivated at either the human host temperature (37°C) or the body louse vector temperature (28°C) was analyzed to identify *B. quintana* niche-specific genes. For this analysis, bacterial transcription was evaluated during the logarithmic phase of growth at both temperatures (Figure 3A). When bacteria were harvested for transcriptional profiling, CFU were enumerated from replicate plates, to ensure that the bacteria were in the logarithmic growth phase (Figure 3B).

**Figure 4:**
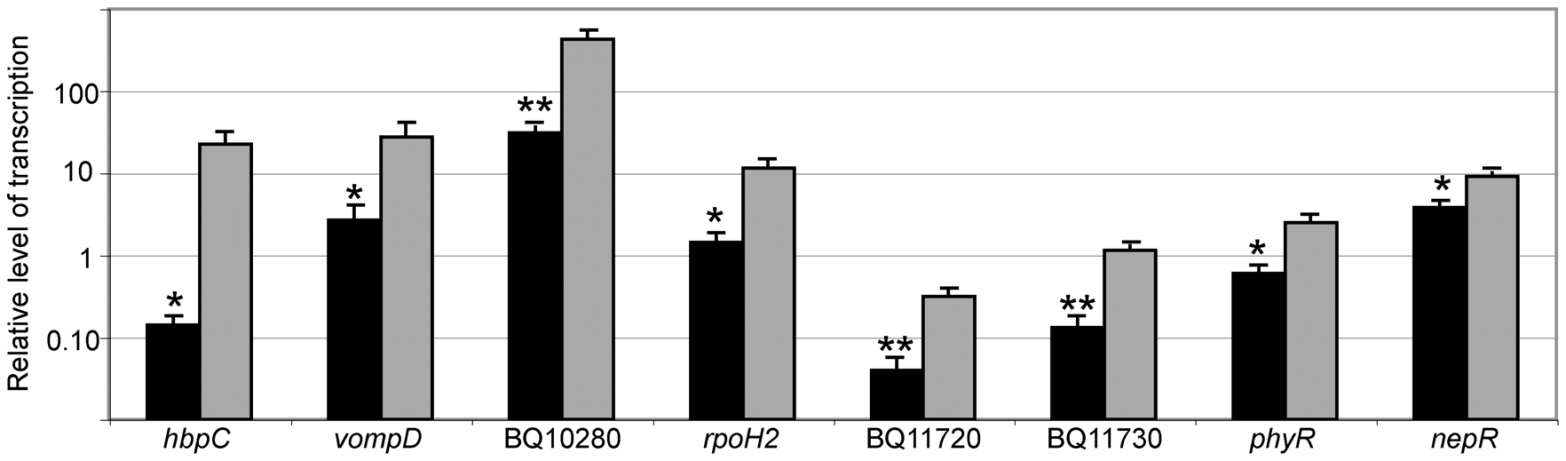
RT-qPCR quantification of *B. quintana* transcription corroborates microarray data for temperature-regulated genes. Transcription of select genes up-regulated at 28°C by microarray analysis was analyzed by RT-qPCR at 37°C (black) and 28°C (gray) to validate the microarray results. Transcript level was normalized to the *B. quintana* reference gene, *purA*. Error bars indicate standard errors of the mean. *, *P*≤0.05; **, *P*≤0.01 by Student's *t* test, comparing the relative level of transcription at 37°C and 28°C for each gene.

Sixty-eight genes were differentially expressed at 37°C versus 28°C by SAM analysis, from replicate time courses (Table 1). Of the temperature-responsive genes, 56 had increased transcription at 28°C, and 12 had decreased transcription at 28°C, compared to 37°C. The results of the microarray transcriptional profiling experiments were validated by RT-qPCR (Figure 4). Three replicate temperature shift experiments were performed, and transcription was analyzed for eight genes found to be temperature-regulated by microarray. The RT-qPCR analysis corroborated the findings of the microarray experiments, demonstrating that the level of transcription of each gene was significantly different at 28°C compared to 37°C (Figure 4).

**Table 1:** *Bartonella quintana* genes differentially expressed at 37°C compared with 28°C.

Gene ID	Description	Name	28°C Log2 ratio	37°C Log2 ratio	Mean local fdr %	Mean Score (d)	Significant Oligos	Fold Change
BQ02410	hemin binding protein c	hpbC	1.62	−3.52	0.02	−5.59	10	−35.25
BQ10280	hypothetical protein		1.51	−2.97	0	−6.41	9	−22.33
BQ00570	hypothetical protein		1.58	−2.50	0	−5.93	2	−16.96
BQ11530	hypothetical protein		1.70	−1.95	0.08	−4.04	4	−12.53
BQ06411	hypothetical protein		1.10	−1.62	0.05	−3.91	6	−6.59
BQ10530	virB secretion system component	virB2	1.05	−1.47	0	−3.70	2	−5.71
BQ10540	virB secretion system component	virB3	0.87	−1.56	0	−3.68	2	−5.38
BQ11720	hypothetical protein		0.63	−1.72	0	−2.76	2	−5.10
BQ11730	hypothetical protein		0.62	−1.59	0	−2.46	7	−4.62
BQ09200	hypothetical protein		0.87	−1.33	0	−3.07	1	−4.59
BQ10980	sensory transduction regulatory protein	phyR	0.38	−1.63	0.78	−2.18	8	−4.00
BQ01390	variable outer membrane protein	vompD	0.40	−1.34	0.36	−1.91	4	−3.34
BQ10550	virB secretion system component	virB4	0.75	−0.98	0	−2.59	8	−3.33
BQ08670	hypothetical protein		0.25	−1.19	0.72	−1.99	6	−2.70
BQ02420	hemin binding protein a	hbpA	1.41	−0.02	5.35	−1.59	7	−2.68
BQ00240	thioredoxin	trxA	0.68	−0.71	1.28	−1.79	2	−2.60
BQ00830	hypothetical protein		0.82	−0.53	0	−2.09	2	−2.56
BQ07681	hypothetical protein		0.70	−0.58	0	−2.11	3	−2.43
BQ11010	hypothetical protein		0.92	−0.32	0	−1.92	1	−2.36
BQ09250	cold shock protein		0.79	−0.43	3.24	−1.71	2	−2.34
BQ08990	regulatory protein		0.60	−0.59	0	−2.59	1	−2.28
BQ06171	hypothetical protein		0.84	−0.32	1.64	−1.78	2	−2.24
BQ10570	virB secretion system component	virB6	0.63	−0.50	1.35	−2.17	6	−2.18
BQ10180	hypothetical protein		0.57	−0.54	1.08	−1.83	3	−2.16
BQ00291	hypothetical protein		0.58	−0.51	0	−3.59	1	−2.14
BQ02770	hypothetical genomic island protein		0.96	−0.11	0.39	−1.85	2	−2.09
BQ06490	transcriptional regulator		0.65	−0.39	0	−2.13	3	−2.05
BQ13370	transmembrane protein		0.68	−0.36	5.11	−1.61	3	−2.05
BQ07300	hypothetical genomic island protein		0.60	−0.43	4.14	−1.64	1	−2.04
BQ10150	hypothetical protein		0.44	−0.58	0.32	−1.95	2	−2.02
BQ06450	hypothetical protein		0.57	−0.43	4.14	−1.65	5	−2.00
BQ09120	integrase recombinase		0.64	−0.33	3.58	−1.69	2	−1.96
BQ11930	hypothetical protein		0.62	−0.35	6.91	−1.54	1	−1.95
BQ06460	hypothetical protein		0.65	−0.30	2.94	−1.72	6	−1.94
BQ07302	hypothetical protein		0.49	−0.39	0.88	−1.82	1	−1.84
BQ01331	hypothetical protein		0.60	−0.26	0.07	−1.88	1	−1.82
BQ11710	biopolymer transport exbB protein	exbB	0.44	−0.41	3.58	−1.68	6	−1.81
BQ11450	hypothetical protein		0.35	−0.50	2.09	−1.74	1	−1.80
BQ12140	ferredoxin II	fdxA	0.27	−0.57	5.96	−1.57	1	−1.79
BQ09410	hypothetical protein		0.43	−0.37	2.60	−1.72	3	−1.74
BQ05180	hypothetical protein		0.84	0.05	4.62	−1.62	1	−1.73
BQ10790	phosphoglucomutase	pgm	0.51	−0.19	2.65	−1.72	5	−1.63
BQ08710	DNA uvrDDNA helicase II	uvrD	0.43	−0.23	2.59	−1.75	5	−1.57
BQ01080	heme exporter protein A	ccmA	0.41	−0.23	1.76	−1.77	4	−1.55
BQ02611	hypothetical protein		0.48	−0.13	6.66	−1.67	1	−1.52
BQ06800	glutathione reductase	gor	0.31	−0.25	0	−1.95	1	−1.47
BQ01070	heme exporter protein B	ccmB	0.40	−0.13	5.65	−1.74	1	−1.44
BQ03390	transcriptional regulator ompR	ompR	0.36	−0.17	6.23	−1.56	2	−1.44
BQ05580	putative integrase dna protein		0.37	−0.10	6.88	−1.54	1	−1.39
BQ06200	glutamate racemase	murI	0.42	0	6.66	−1.55	1	−1.34
BQ10290	probable surface protein		0.20	−0.14	0	−3.08	1	−1.27
BQ07580	exopolyphosphatase		0.38	0.03	6.73	−1.54	1	−1.27
BQ05030	hypothetical protein		0.25	−0.03	4.93	−1.61	5	−1.21
BQ05940	nitrogenase cofactor synthesis protein	nifS1	0.56	0.29	6.40	−1.55	1	−1.21
BQ08870	cell division protein	ftsW	0.03	0.15	3.48	−1.67	1	−0.92
BQ03510	chorismate synthase	aroC	−0.14	0.06	0	−1.77	1	−0.87
BQ08760	DNA ligase	ligA	−0.33	0.26	0	2.20	1	1.50
BQ03710	phosphatase		−0.56	0.09	0.31	1.83	1	1.57
BQ02310	ABC transporter permease protein		−0.51	0.15	1.59	1.76	1	1.58
BQ12210	transport protein transmembrane		−0.37	0.31	0.34	1.83	1	1.61
BQ00450	hypothetical protein		−0.53	0.30	0.35	1.86	5	1.78
BQ12890	SUN protein FMU protein	sun2	−0.35	0.51	0.24	1.89	6	1.81
BQ00840	nicotinate phosphoribosyltransferase	pncB	−0.77	0.11	0	1.92	4	1.84
BQ11820	tolB protein	tolB	−0.90	0.08	0.31	1.87	3	1.97
BQ01770	ABC transporter, periplasmic binding protein		−0.71	0.31	0.30	2.09	5	2.02
BQ01780	ABC transporter, permease protein		−0.43	0.62	0.01	2.03	10	2.07
BQ12900	heat shock protein		−0.54	0.54	0.79	1.81	2	2.11
BQ00600	heat shock protein dnaJ	dnaJ1	−1.07	0.39	0.16	1.90	8	2.75

**Figure 5:**
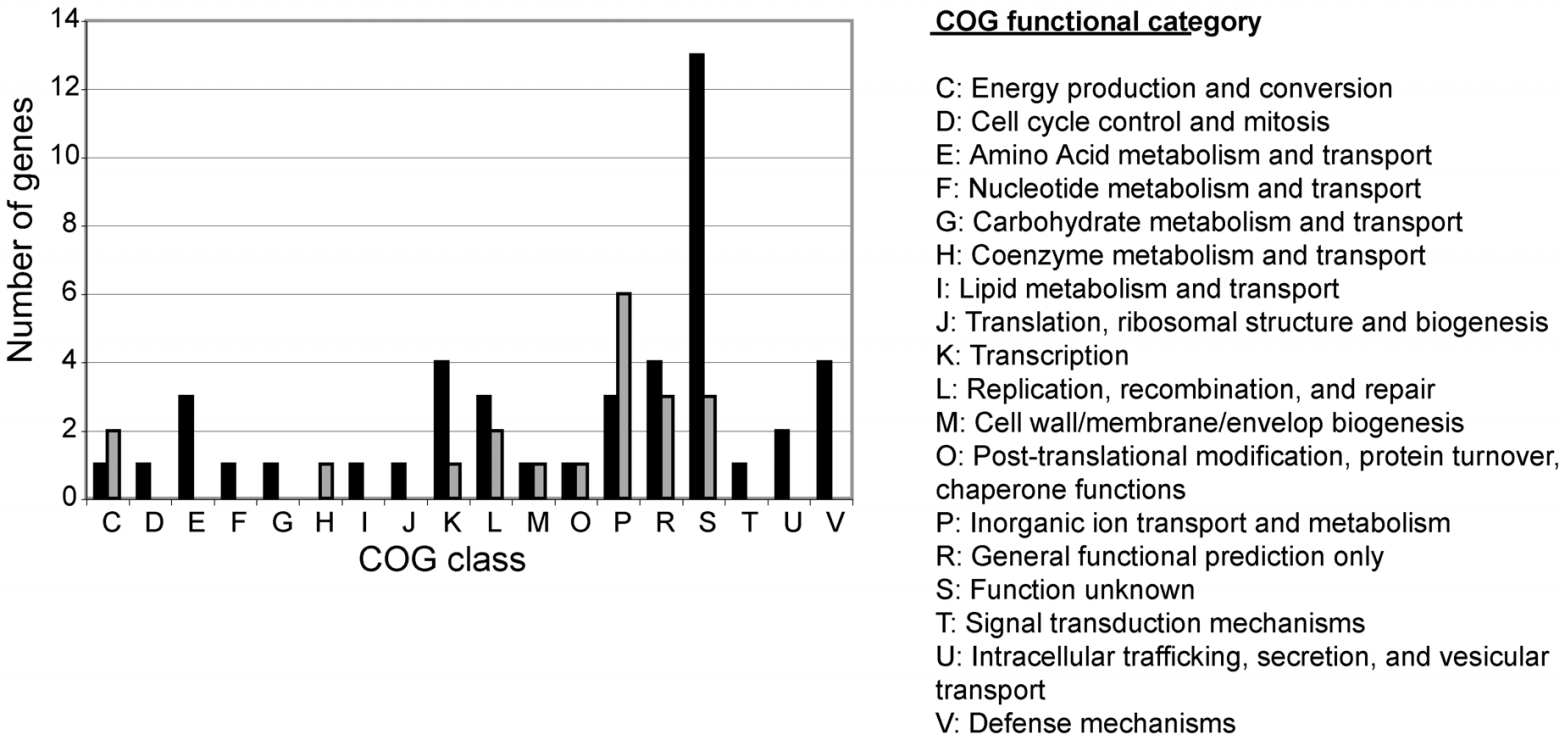
*B. quintana* genes up-regulated at 28°C are overrepresented in several COG functional categories. The graph shows the COG classification of each gene that was significantly up- or down-regulated in *B. quintana* grown at 28°C, from microarray analysis (Table 1). Genes with increased transcription at 28°C are represented by black bars; genes with decreased transcription at 28°C are represented by gray bars. Of the categories with attributable function, there is an overrepresentation of up-regulated genes in the transcription, signal transduction, intracellular trafficking/secretion/vesicular transport, and defense mechanisms in *B. quintana* grown *in vitro* on agar, at the arthropod vector temperature of 28°C. The greatest number of genes down-regulated at 28°C are in the inorganic ion transport and metabolism category.

We classified the temperature-responsive genes identified by our microarray analysis into functional categories, based on the classification scheme of the Cluster of Orthologous Groups (COG) database [29] (Figure 5). The temperature-regulated genes within COG functional category P (inorganic ion transport and metabolism) were of particular interest because of their potential role in *B. quintana* hemin metabolism and detoxification. Hemin and hemoglobin are the only iron sources that *Bartonella* can metabolize [30], making acquisition and metabolism of these nutrients essential for *B. quintana* survival. A major difference between the host and vector environments is the amount of free hemin available. The human bloodstream is extremely hemin restricted, whereas toxic levels of hemin are present in the body louse alimentary tract. Hemin can produce reactive oxygen molecules that are potentially toxic [31]. *Bartonella* is unique in its ability to survive exposure to hemin concentrations that are typically bactericidal (>1 mM) [30], [32], [33]. We identified four hemin-related proteins in COG functional category P that are up-regulated at 28°C: hemin binding protein A (*hbpA*), hemin binding protein C (*hbpC*), and heme exporter protein A and B (*ccmA, ccmB*) (Table 1). These gene products likely are involved in facilitating survival of *B. quintana* when exposed to toxic hemin concentrations in the body louse.

As their name suggests, the hemin binding proteins (Hbp) bind hemin [34]. Previous analysis of temperature-specific transcription of the five *hbp* family genes in *B. quintana* by Battisti, *et al.* [35], identified *hbpC* as temperature-responsive. Similar to what is observed in *B. quintana, B. henselae hbpC* displays increased expression at 28°C versus 37°C when cultivated on chocolate agar [36]. In *B. henselae*, up-regulated expression of *hbpC* at arthropod temperature ameliorates the antibacterial toxicity of the concentrated hemin in the arthropod gut [36]. Thus, the significant up-regulation of *hbpC* appears to be part of the critical hemin detoxification response in *Bartonella* species during adaptation to the arthropod niche. Of note, the greatest number of genes down-regulated at 28°C are also in COG functional category P (Figure 5). In addition to up-regulation of genes that ameliorate hemin toxicity at 28°C, it would be critical to down-regulate any *B. quintana* genes that mediate binding and uptake of the stringently-sequestered hemin in the human bloodstream.

Also prominent among the temperature-regulated genes are some components of the VirB T4SS, a second T4SS (in addition to Trw) in *B. quintana*. The VirB T4SS apparatus is involved in the injection of effector proteins into host cells [37], [38], and appears to have a different function from the Trw T4SS [37]. Of note, *virB2*, *virB3*, *virB4*, and *virB6* are highly up-regulated at 28°C (Table 1); in contrast, *virB8*-*11* are growth-phase regulated (d-score 3.3–3.8) but not temperature regulated, so perhaps multiple environmental cues are integrated before producing the fully functional VirB secretion complex encoded on adjacent but distinct operons. The Trw T4SS components are growth-phase regulated, supporting the differential function and responsiveness to environmental cues for these two *B. quintana* T4SS.

The expression of two response regulators (COG functional category T), encoded by *B. quintana phyR* (BQ10980) and *ompR* (BQ03390), were found to be temperature-responsive. Expression of *phyR* was increased 4-fold at 28°C versus 37°C (Table 1, Figure 4). *B. quintana* PhyR is a positive regulator of RpoE (unpublished data); *B. quintana* RpoE is an alternative sigma factor that is involved in transcription of genes necessary for survival in the high hemin environment of the body louse gut (unpublished data). As predicted, we found that *phyR*, the positive regulator of *rpoE*, is one of the most highly transcribed genes at body louse temperature. Expression of the response regulator *ompR* also was increased at 28°C (Table 1). In *B. henselae, ompR* is transcribed in response to contact with human endothelial cells [39], and OmpR has been shown to be involved in *B. henselae* invasion of human endothelial cells *in vitro* [40]. Our observation that *ompR* transcription is temperature regulated suggests that OmpR is involved in priming human endothelial cell invasion by *B. quintana* during the transition from body louse to mammalian host. Our data also suggest niche-specific roles for other, less-studied transcriptional regulators (COG functional category K) in *B. quintana*, such as BQ08990 and BQ06490. BQ08990 has homology to the ArsR family of transcriptional regulators, which has a role in sensing environmental metal concentrations, and in the induction of pathogenicity in *Bacillus anthracis* and *Streptococcus mutans* [41], [42], [43]. BQ06490 has homology to the AsnC transcriptional regulators that are typically involved in environmentally-cued induction of alternative amino acid metabolic pathways [44]. It thus appears that ambient temperature drives niche adaptation by controlling expression of several transcriptional regulators (4 genes of the 37 genes annotated as transcriptional regulators).

### Annotation of unannotated, temperature-responsive *B. quintana* genes reveals potential niche-specific virulence genes

Many of the genes identified as temperature-responsive were unannotated. We reevaluated the annotation of these genes using homology searches. The full-length peptide sequences of the temperature-responsive, unannotated *B. quintana* genes were evaluated using blastp and pHMMER search engines against nr database (http://hmmer.janelia.org/search/phmmer). We annotated 18 genes with E-values of 4.00E-08 or less as putative *B. quintana* homologs. These genes are shown in Table 2. In most cases, these improved gene annotations were corroborated by both the pHMMER and blastp search results.

**Table 2:** Identification of homologs for unannotated, temperature-responsive *Bartonella quintana* genes.

	BlastP			pHMMER		
locus ID	Description	E-value	NCBI accession #	Description	E-value	GI/accession #
BQ00450	zinc metalloprotease	6.00E-77	NP_540932.1	zinc metalloprotease	8.50E-71	306837668
BQ00570	LysM domain/BON superfamily protein	2.00E-52	EHH06667.1	LysM domain/BON superfamily protein	3.00E-47	325291490
BQ00830	-	-	-	inner membrane protein ybaN	1.70E-23	358048936
BQ02770	XRE family transcriptional regulator	4.00E-08	YP_002004972.1	conserved hypothetical protein *Bartonella* sp. AR 15-3	3.90E-09	319405804
BQ05030	glycosyl transferase family protein	6.00E-11	YP_001877749.1	glycosyltransferase sugar-binding protein containing DXD motif	6.20E-11	299133438
BQ06171	PP-loop domain containing protein	2.00E-16	ZP_05779946.1	tRNA 2-thiocytidine biosynthesis protein TtcA	3.10E-12	81648390
BQ06411	-	-	-	similar to ankyrin 2,3/unc44, partial	2.10E-49	115950018
BQ06450	Staphylococcal nuclease homolog	2.00E-63	CBI82181.1	nuclease domain-containing protein	4.10E-29	261758074
BQ06460	uracil-DNA glycosylase	1.00E-82	EHJ97859.1	Uracil-DNA glycosylase	9.30E-79	306840362
BQ08670	-	-	-	PF11015.3 n/a Protein of unknown function (DUF2853) 2 100	9.40E-35	DUF2853
BQ09200	Transglycosylase-associated protein	3.00E-26	YP_002290717.1	transglycosylase-associated protein	3.20E-30	306843863
BQ09410	cation diffusion facilitator family transporter	1.00E-126	ZP_04680778.1	cation diffusion facilitator family transporter	8.40E-117	306844567
BQ10150	trm112p-like family protein	3.00E-17	ZP_08269540.1	Trm112p-like protein	6.20E-11	PF03966.11
BQ10180	SH3 type 3 domain-containing protein	3.00E-09	EHK80050.1	bacterial SH3 domain protein	1.00E-12	342212994
BQ10280	Inducible *Bartonella* autotransporter	4.00E-131	CBI80621.1	CAMP-like factor autotransporter	4.70E-267	56684460
BQ10290	inducible *Bartonella* autotransporter	5.00E-122	CBI80621.1	CAMP-like factor autotransporter	<1.00E-300	56684460
BQ11930	Sel1 repeat-containing protein	8.00E-53	ZP_04681125.1	Sel1 domain protein repeat-containing protein	6.70E-63	163800487
BQ11720	-	-	-	PF05957.8 Bacterial protein of unknown function (DUF883)	1.40E-18	DUF883

One previously unannotated gene of particular interest was gene BQ00450, which was up-regulated at 37°C (Table 1). Our updated annotation classified this gene as a putative zinc metalloprotease (Table 2). Zinc metalloproteases are found in pathogenic bacteria and have been implicated in bacterial invasion and pathogenicity in *Pseudomonas aeruginosa, Vibrio cholerae,* and *Bacillus anthracis* [45]. These metalloproteases act to cleave immune effector proteins and to remodel the niche for bacterial attachment. It is possible that BQ00450 has a similar role in *B. quintana* colonization of the human host.

We annotated the genes BQ10280 and BQ10290 as putative autotransporters, and identified orthologous genes in many other *Bartonella* spp. (all give blastp hits with E-value <1E-129) (Table 2). Both of these putative autotransporter genes were highly up-regulated at 28°C (Table 1; Figure 4), and their genomic placement suggests that they could be co-transcribed as an operon. Autotransporters serve a number of virulence functions in bacteria; of particular interest, they are involved in adhesion [46], [47], [48] and in biofilm formation [49]. *B. quintana* adheres to body louse gut epithelial cells [50], and the bacteria form a biofilm-like structure within the louse feces [21], but the *B. quintana* proteins and molecular mechanisms involved in both of these processes are unknown. These autotransporters, BQ10280 and BQ10290, which are highly expressed at the vector temperature, could be involved in *B. quintana* adhesion or biofilm formation in the body louse gut.

### A purine-rich, temperature-responsive, putative promoter motif is identified for genes up-regulated at body louse temperature (28°C)

**Figure 6:**
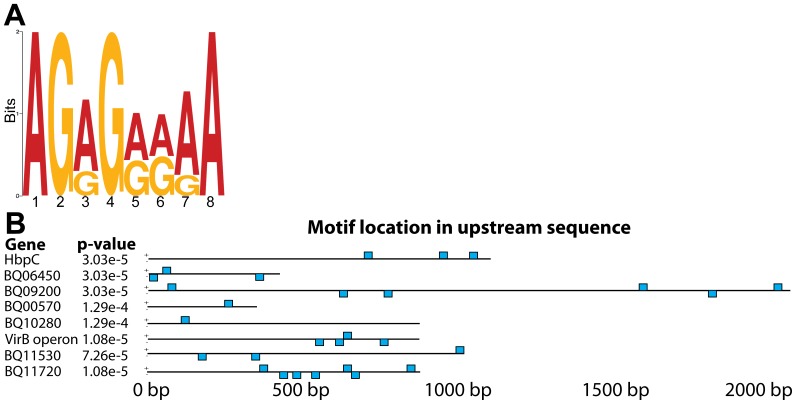
MEME searching identifies an overrepresented, purine-rich motif upstream of *B. quintana* genes up-regulated at 28°C (A). Sequence logo of the top scoring MEME result for the top 11 regulated genes, by SAM score; and **(B)** position and scoring of motif sites (p-value threshold <1e-3) in upstream sequences. The motif is present in upstream sequences for 8 of the top 11 genes, often with multiple instances, as shown by the blue block diagram depicting motif position within upstream sequences.

We analyzed the upstream intergenic sequences of the differentially-regulated, temperature-responsive genes to identify motifs that correlate with temperature-dependent changes in expression, using the MEME algorithm. The temperature-responsive genes up-regulated at 37°C did not produce any significant MEME results. MEME analysis of all the 28°C-specific genes, or just the upstream noncoding regions of the eleven genes most highly transcribed at 28°C, returned a single motif with E-value <0.1. This 8-mer motif was purine-rich (‘AGRGRRRA’), with an E-value of 8.3×10^−3^. Additionally, variants of this motif repeatedly scored well over a range of motif-length input parameters, from 6-mers to 12-mers (Figure 6A). The identified motif was present 35 times in 10 of the 11 upstream regions. For example, this motif was repeated three times upstream of *hbpC* and four times upstream of genes in the *virB* T4SS operon (Figure 6b).

### Quantification of *in vivo* transcripts in *B. quintana* from infected body lice by RT-qPCR corroborates up-regulated genes identified by RT-qPCR and microarray from *B. quintana* grown *in vitro* on agar at 28°C

From the *in vitro* microarray analysis, we identified a number of genes whose transcription was increased at 28°C and thus could represent genes critical for *B. quintana* colonization of the body louse vector. *In vivo* analysis of *B. quintana* transcription was performed to corroborate our *in vitro* microarray data. Female lice in a colony established from body lice removed recently from an infested person were used for the *in vivo* experiments. These lice were fed only human blood, through an artificial membrane-rearing system [20], instead of using the Culpepper body louse laboratory strain that was adapted decades ago to feed only on live rabbits [51]. The artificial membrane model is a more appropriate model, because the rabbit does not sustain *B. quintana* bacteremia and is not a relevant host for transmitting *B. quintana* to human lice. The lice were infected by feeding for 24 hours on a *B. quintana*-inoculated human blood meal, and then were fed subsequently on uninfected human blood.

**Figure 7:**
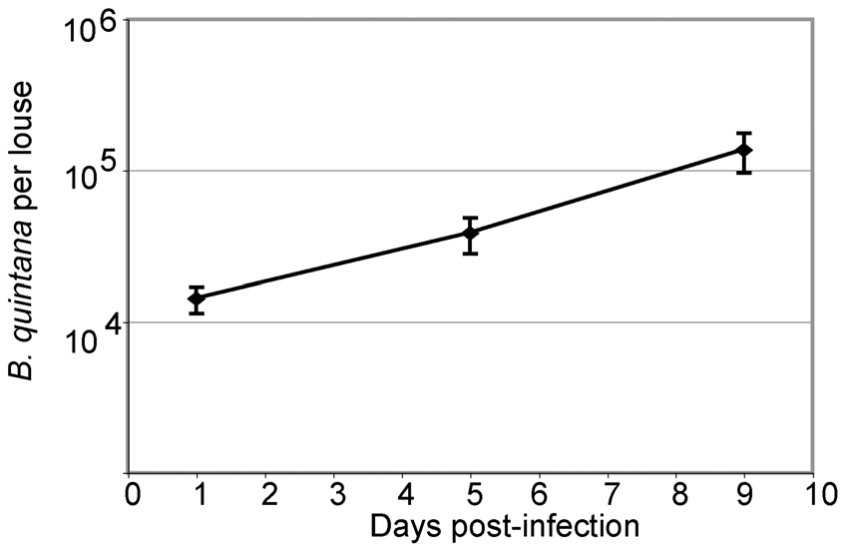
The number of *B. quintana* per body louse increases over time during *in vivo* infection. The number of *B. quintana* bacteria per louse was determined by real-time PCR analysis of DNA isolated from infected body lice. At 1 day post-infection (dpi), there were approximately 1.42×10^4^ ±2.83×10^3^ *B. quintana* per louse; at 5 dpi, 3.82×10^4^ ±1.02×10^4^ *B. quintana* per louse; and at 9 dpi, 1.36×10^5^ ±4.00×10^4^ *B. quintana* per louse. These findings corroborate the quantification of *B. quintana* in experimentally infected body lice reported by Seki *et al.,* 2007. The average of data from three separate experiments is shown; error bars represent the standard errors of the mean.

We first established that the number of *B. quintana* bacteria per louse increased over the course of the infection, by performing quantitative analysis of *B. quintana* proliferation in the infected body lice, using real-time PCR. Figure 7 documents infection of the body lice with viable, replicating *B. quintana*. Similar rates of *B. quintana* replication were observed in our study and the previous work by Seki, *et al*. [21].

**Figure 8:**
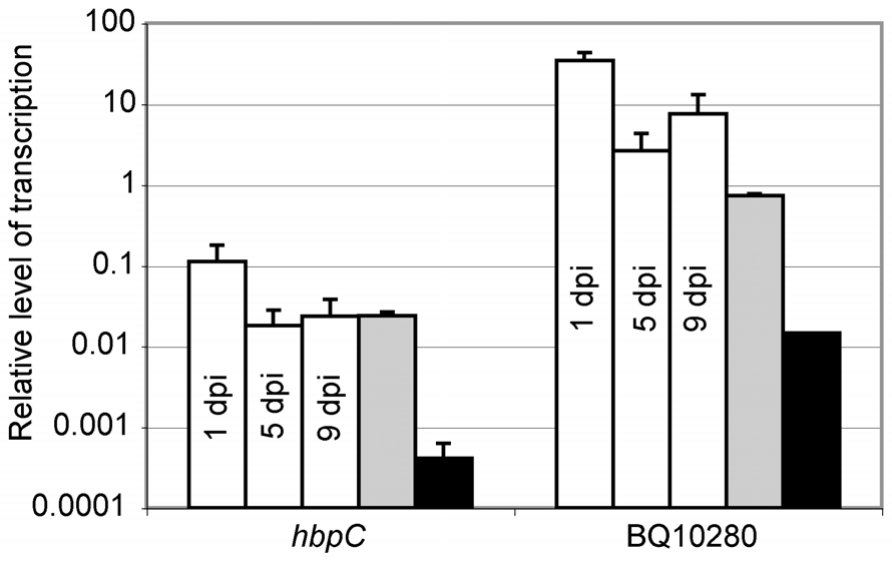
Transcription of *hbpC* and BQ10280 *in vivo* corroborates transcription results *in vitro* at 28°C. *In vivo* transcription of *hbpC* and BQ10280, genes determined to be highly expressed *in vitro* at 28°C by microarray, was analyzed in *B. quintana*-infected body lice (white bars) at 1, 5, and 9 days post-infection (dpi) by RT-qPCR. The *in vitro* transcription of *hbpC* and BQ10280 in *B. quintana* grown *in vitro* on chocolate agar at 28°C (gray bars) or 37°C (black bars) also was evaluated by RT-qPCR. Transcript level was normalized to *B. quintana* 16S rRNA. The relative level of *hbpC* and BQ10280 transcript in infected body lice was similar to that observed during *in vitro* growth of *B. quintana* at 28°C. The average of data from three separate experiments is shown; error bars represent the standard errors of the mean.

RNA was isolated from the lice 24 hours after feeding on the *B. quintana*-containing human blood meal, and at five and nine dpi for transcriptional analysis. We quantified the expression of two *B. quintana* genes (*hbpC* and BQ10280) in body lice by RT-qPCR. We previously found that these two genes were highly up-regulated in *B. quintana* grown *in vitro* on agar at 28°C, using microarray transcriptional profiling (Figure 4). The relative level of transcription of *hbpC* and BQ10980 in lice was similar to that observed when the *B. quintana* were cultivated *in vitro* on chocolate agar plates at 28°C, and was greater than that observed when the bacteria were cultivated on chocolate agar plates at 37°C (Figure 8). Transcription of both genes was greatest at 1 dpi, suggesting that HbpC and BQ10280 have an important role in initial vector colonization. Although the transcription of these two genes at 28°C was similar *in vivo* and *in vitro*, there are likely other *B. quintana* genes that are up- or down-regulated by stimuli found only *in vivo*, within the body louse. These data provide the first insight into the *B. quintana* transcriptional program within the body louse environment, leading the way to subsequent *in vivo* studies that can define the mechanisms by which *B. quintana* transitions between the human host and the arthropod body louse vector.

## Conclusions

*B. quintana* must survive and proliferate within the body louse vector, as well as the human host, during the course of its infectious cycle. Each of these niches presents the *B. quintana* bacteria with unique nutritional and environmental conditions. To begin to understand how *B. quintana* adapts to each environment, we analyzed global transcription in bacteria grown at temperatures corresponding to the human host (37°C) or the body louse vector (28°C). We observed unique patterns of gene expression at each of these two niche-associated temperatures. These genes included temperature-specific virulence factors with known or predicted roles in secretion, iron binding and transport, and regulation of transcription. For some of the genes that were only described as encoding ‘hypothetical proteins,’ we improved the annotation and identified additional, potential virulence genes whose expression is temperature-regulated. Upstream of some of the genes that were up-regulated at 28°C, we identified a conserved, purine-rich motif that could permit coordinate transcription of temperature-regulated, niche-specific *B. quintana* genes.

Our *in vitro* whole genome transcriptional profiling microarray data from *B. quintana* grown on agar at 28°C, were corroborated *in vivo* using RT-qPCR to document up-regulation of mRNA expression in the body louse for two select *B. quintana* genes. For this *in vivo* quantification of *B. quintana* mRNA, we used a novel model for body louse infection that recapitulates the natural route of infection of body lice with *B. quintana*. The louse infection model utilizes an artificial membrane-feeding system [20] that enabled us to feed lice on human blood inoculated with *B. quintana.* Future experiments will utilize whole transcriptome analysis to identify differentially up- and down-regulated *B. quintana* genes in the body louse, as well as the unique environmental signals to which these genes are responsive. From the perspective of transcriptional regulation, we found that the transition from mammalian host to arthropod vector temperature principally involves deployment of different hemin binding systems and the preparation of export systems to adapt to the new niche. During this work, we have developed important tools (*in vitro* whole genome *B. quintana* DNA microarray, and *in vivo* body louse infection with *B. quintana*) that provide a new understanding of *B. quintana* host and vector adaptation and will allow further study of the host-vector relationship.

## Supporting Information

Table S1 **Oligonucleotide primers used in this study.**
(DOCX)

Table S2 ***B. quintana*** **genes that are transcriptionally responsive to the transition from logarithmic growth phase to stationary/death phase.**
(DOCX)
