# Supplementary material for: Bartonella quintana Deploys Host and Vector Temperature-Specific Transcriptomes
Source: PLoS One. 2013 Mar 12;8(3):e58773. doi: 10.1371/journal.pone.0058773 (PMC3595295; doi:10.1371/journal.pone.0058773)
Supplement: Table S1 — Oligonucleotide primers used in this study. (DOCX) [file pone.0058773.s001.docx]

## Table S1. Oligonucleotide primers used in this study

| **Primer** | **Sequence** | **Purpose** |
| --- | --- | --- |
| SA090 | ggattgtacgtggcgtcttt | RT-qPCR *purA*, forward primer |
| SA091 | aatggaccttctccaacacg | RT-qPCR *purA*, reverse primer |
| SA082 | atgaatatgaaatggttaataacgg | RT-qPCR *hbpC*, forward primer |
| SA083 | ccgttagcgagaatattcatctt | RT-qPCR *hbpC*, reverse primer |
| SA346 | gcaggcaaggcgaacgt | RT-qPCR *vompD*, forward primer |
| SA347 | tcatgtttgggccaccagta | RT-qPCR *vompD*, reverse primer |
| SA128 | TTTTTGTTGATTGCCGTTGA | RT-qPCR *phyR*, forward primer |
| SA129 | GGGTTCGTGCTATCCCTACA | RT-qPCR *phyR*, reverse primer |
| SA078 | tattttacgaaattggtcaatcgtt | RT-qPCR *rpoH2*, forward primer |
| SA079 | tatcagagctagaaaaacggaaatc | RT-qPCR *rpoH2*, reverse primer |
| SA224 | agatgatcttctcggggtca | RT-qPCR *nepR*, forward primer |
| SA225 | tcaaaccttttctgcattgttt | RT-qPCR *nepR*, reverse primer |
| SA096 | cagctcgtgtcgtgagatgt | RT-qPCR 16S rRNA, forward primer |
| SA097 | cagagtgcaatccgaactga | RT-qPCR 16S rRNA, reverse primer |
| SA354 | cgcaatgaaatttccggtat | RT-qPCR BQ11720, forward primer |
| SA355 | gaataaacccaacccctgct | RT-qPCR BQ11720, reverse primer |
| SA356 | acgtttatcgctcccctttt | RT-qPCR BQ11730, forward primer |
| SA357 | agaaatcggccaacacaaac | RT-qPCR BQ11730, reverse primer |
| SA249 | aatggagacacgtcctacgg | RT-qPCR BQ10280, forward primer |
| SA250 | ctcctgaaagttcgctttgc | RT-qPCR BQ10280, reverse primer |
| Seki_1 | GCCGCCTTCGTTTCTCTTTC | *B. quintana* quantification, forward primer |
| Seki_2 | AGTGTCTTCCTTAAAGTCCCAAAG | *B. quintana* quantification, reverse primer |
