# Supplementary material for: Bartonella quintana Deploys Host and Vector Temperature-Specific Transcriptomes
Source: PLoS One. 2013 Mar 12;8(3):e58773. doi: 10.1371/journal.pone.0058773 (PMC3595295; doi:10.1371/journal.pone.0058773)
Supplement: Table S2 — B. quintana genes that are transcriptionally responsive to the transition from logarithmic growth phase to stationary/death phase. (DOCX) [file pone.0058773.s002.docx]

**Table S2. *B. quintana* genes that are transcriptionally responsive to the transition from logarithmic growth phase to stationary/death phase.**

| **Gene ID** | **Gene Name** | **Name** | **Fold Change*** |
| --- | --- | --- | --- |
| BQ11580 | hypothetical intracellular effector | yopP | 2.41 |
| BQ12950 | carbonic anhydrase protein |  | 2.40 |
| BQ11110 | hypothetical protein |  | 2.37 |
| BQ09900 | hypothetical protein |  | 2.26 |
| BQ11120 | hypothetical protein |  | 2.18 |
| BQ02790 | Phosphoserine aminotransferase | serC | 2.17 |
| BQ11350 | hypothetical protein |  | 2.13 |
| BQ10950 | hypothetical protein |  | 2.11 |
| BQ10620 | virB11 protein homolog | virB11 | 2.04 |
| BQ02400 | hypothetical protein |  | 2.04 |
| BQ12600 | trwI1 protein_10163 | trwI1 | -2.97 |
| BQ04040 | Aminopeptidase N_3556 | pepN | -2.97 |
| BQ06720 | hypothetical protein |  | -2.98 |
| BQ12590 | trwJ1 protein | trwJ1 | -2.99 |
| BQ13530 | Chromosome partitioning protein | parB | -2.99 |
| BQ07520 | hypothetical protein |  | -2.99 |
| BQ10020 | Multidrug resistance protein | vceA | -3.00 |
| BQ08730 | sco1 family protein | sco1 | -3.00 |
| BQ08430 | Hemin binding protein E | hbpE | -3.00 |
| BQ08750 | Aminopeptidase P protein |  | -3.01 |
| BQ10720 | hypothetical protein |  | -3.01 |
| BQ04920 | Pyruvate dehydrogenase E1 component beta subunit | pdhB | -3.02 |
| BQ09890 | omp43 precursor | omp43 | -3.02 |
| BQ10750 | Chaperonin protein groEL | mopA | -3.02 |
| BQ11750 | Cell division protein | ftsH | -3.02 |
| BQ08460 | hypothetical protein |  | -3.03 |
| BQ09280 | Thioredoxin reductase | trxB | -3.03 |
| BQ13330 | pH adaptation potassium efflux system E | phaE | -3.03 |
| BQ04910 | Pyruvate dehydrogenase E1 component alpha subunit | pdhA | -3.04 |
| BQ12560 | trwL8 protein | trwL8 | -3.05 |
| BQ03220 | sohB Protease | sohB | -3.06 |
| BQ08310 | Amino acid permease_6924 | gltJ | -3.06 |
| BQ09810 | hypothetical protein |  | -3.06 |
| BQ13520 | Hydroxyacylglutathione hydrolase | gloB | -3.06 |
| BQ05970 | hypothetical protein |  | -3.07 |
| BQ12490 | trwL1 protein | trwL1 | -3.07 |
| BQ12510 | BQ12510trwL3trwL3_protein_10114 | trwL3 | -3.08 |
| BQ05950 | ABC transporter subunit |  | -3.09 |
| BQ00540 | hypothetical protein |  | -3.10 |
| BQ10800 | hypothetical protein |  | -3.13 |
| BQ09490 | hypothetical protein |  | -3.14 |
| BQ09170 | DNA primase | dnaG | -3.15 |
| BQ12530 | trwL5 protein | trwL5 | -3.15 |
| BQ08780 | Competence lipoprotein comL precursor | comL | -3.18 |
| BQ12840 | SurF1 family protein | surF1 | -3.18 |
| BQ04430 | Amidophosphoribosyltransferase precursor | purF | -3.20 |
| BQ12040 | hypothetical protein |  | -3.21 |
| BQ12360 | hypothetical protein |  | -3.22 |
| BQ06850 | Citrate synthase | gltA | -3.25 |
| BQ00870 | Cytidylate kinase | cmk | -3.26 |
| BQ05450 | hypothetical protein |  | -3.26 |
| BQ08360 | hypothetical protein |  | -3.27 |
| BQ00310 | Folylpolyglutamate synthase | folC | -3.28 |
| BQ08320 | Amino acid permease | gltK | -3.28 |
| BQ01250 | 30s ribosomal protein | rpsU | -3.29 |
| BQ03580 | hypothetical protein |  | -3.29 |
| BQ05310 | Acyl carrier protein | acpP2 | -3.32 |
| BQ05330 | hypothetical protein |  | -3.33 |
| BQ07410 | hypothetical protein |  | -3.35 |
| BQ04600 | hypothetical protein |  | -3.39 |
| BQ03400 | hypothetical protein |  | -3.40 |
| BQ09260 | Aspartate aminotransferase A | aatA | -3.41 |
| BQ04050 | DNA polymerase, bacteriophage type |  | -3.43 |
| BQ01870 | Preprotein translocase secA subunit | secA | -3.43 |
| BQ02190 | hypothetical protein |  | -3.44 |
| BQ09040 | hypothetical protein |  | -3.45 |
| BQ07710 | hypothetical protein |  | -3.46 |
| BQ02570 | DNA mismatch repair protein | mutL | -3.46 |
| BQ09180 | hypothetical protein |  | -3.47 |
| BQ09140 | Outer membrane protein |  | -3.47 |
| BQ09380 | hypothetical protein |  | -3.49 |
| BQ12620 | trwJ2 protein | trwJ2 | -3.50 |
| BQ02530 | hypothetical protein |  | -3.50 |
| BQ07600 | hypothetical protein |  | -3.50 |
| BQ06280 | Cold shock protein |  | -3.52 |
| BQ07390 | Phosphoribosylglycinamide formyltransferase | purN | -3.55 |
| BQ03780 | hypothetical protein |  | -3.57 |
| BQ04440 | Colicin v production protein | cvpA | -3.58 |
| BQ10070 | hypothetical protein |  | -3.61 |
| BQ12480 | korA protein | korA | -3.61 |
| BQ01660 | Outer membrane lipoprotein precursor |  | -3.62 |
| BQ12570 | trwM protein | trwM | -3.73 |
| BQ12790 | DnaJ related protein |  | -3.78 |
| BQ03080 | hypothetical protein |  | -3.80 |
| BQ03190 | hypothetical protein |  | -3.81 |
| BQ08500 | hypothetical protein |  | -3.83 |
| BQ00690 | Polypeptide deformylase_695 | def | -3.92 |
| BQ04750 | hypothetical protein | tatB | -3.93 |
| BQ01860 | Iron response regulator | fur2 | -3.93 |
| BQ04930 | Dihydrolipoamide acetyltransferase E2 | pdhC | -3.94 |
| BQ00010 | hypothetical protein |  | -4.15 |
| BQ08290 | Exodeoxyribonuclease III | xthA1 | -4.19 |
| BQ03790 | hypothetical protein |  | -5.04 |

*****The fold increase represents the fold difference in *B. quintana* transcription in stationary phase, compared with logarithmic growth phase.
